# Miscellaneous Contributions to Pathology and Therapeutics

**Published:** 1844-10-01

**Authors:** 


### Miscellaneous Contributions to Pathology and Therapeutics. By *James Richard Smyth*, M.D. Pp. 341. London, 1844.

The Essays which this volume contains originally appeared in the weekly medical journals, and are not, in our opinion, of a sufficiently original or important character to justify their republication in the present form. If, indeed, we are to admit as a necessity, as it has become almost a rule of modern practice, that every practitioner should have, as part of his armoury, his own work gracing the shelves of his library, then we are free to allow that we have perused many works containing fewer facts and less interesting observations than the present. But to the existence of any such necessity, at least as far as readers are concerned, we are disposed to demur; and the great utility of the medical periodicals consists in the ready vehicle they form for the reception of observations too important to be lost, and yet not sufficiently so to appear in a more substantive form. In this way channels for the circulation of facts and opinions are opened to an extent attainable by no other means, while equal opportunities being afforded for corroboration or refutation on the part of others, whatever of real value the contributions may contain is eventually elicited, and an equivalent reputation cheerfully accorded to their author by his fellows. Great as these benefits doubtless are they are not unattended by disadvantages, for the facility of "rushing into print" possessed in medical which does not prevail in general literature, frequently gives rise to the production of many crude and worthless communications, and to many others, to which although such character cannot attach, would still have much benefited by their publication being delayed until a more matured judgment, and more extensive experience had been brought to bear upon their investigation. We do not apply these latter observations to Dr. Smyth's papers, which are very interesting ones, although not possessing sufficient stamen to render their collection into an expensive volume, a desirable proceeding.

The subjects treated of by the author are Rickets, Chronic Hydrocephalus, Cerebral Auscultation, Impotence, Hæmoptysis, Rheumatic Pericarditis, and False Aneurysm—and we may extract a few of his observations upon some of these.

*Rickets.*—In the Preface the author observes, "It will be seen that I have attributed the origin and phenomena of that affection chiefly to infantile indigestion; and that enlargement, in a greater or less degree, of the liver is a prominent feature of its pathology; and requires to be duly considered in the treatment of the affection. It will also be seen that I look upon rickets and scrofula as diseases identical in their causes and natures, and demanding for their cures similar remedies and modes of treatment. Next to the subject of rickets I have treated of that of hydrocephalus, as I consider the two maladies, and also that of scrofula, as manifestations of the same morbid diathesis."

The great influence which the condition of the liver exerts upon the economy in this disease is alluded to in other passages, as in the following:—

"My experience, I acknowledge, does not enable me to say to what extent enlargement of the liver, and more or less prolonged defect or vitiation of the biliary secretion in children, deranging and impairing the processes of infantile digestion and hæmatosis, may be connected with the proximate cause and early phenomena of rickets; but I could not avoid observing, during the treatment of the first two cases of the subject of this paper, in which great enlargement of the liver was present, that the return to a more healthy size of that viscus, and improvement of the general condition of each individual, and marked amendment of the rickety symptoms, were perfectly simultaneous. * *

Is it unreasonable to suppose that if the liver should become diseased or defective in function, and continue for any lengthened time during infancy, the period of most tender growth and conformation of the individual, it must affect, in some very obvious manner the processes of assimilation and healthy organic formation throughout the entire system? Look at a child, the subject of slight taint, or what you might consider as the state of commencing rickets. Criticise its entire condition. Does it not bear a strong resemblance, in many points, to that of an adult whom one would suspect to be labouring under some chronic concealed affection of the liver? So has it, in fact, not unfrequently struck my observation. At all events I am firmly of opinion that the practitioner who will view the infantile affection now under consideration as one simply of hepatic disease, with more or less aggravated derangement of all the digestive functions, and so apply his remedies judiciously in accordance, will be he who is most likely to benefit, and in all respects improve, the condition of his patient." 20.

That the same exciting causes which so generally prevail among the children of the lower classes may sometimes produce scrofula, at others hydrocephalus, and at others rickets, there can be no doubt; but we are far from believing that these affections are hence necessarily identical, or to be relieved always by the same proceedings. For all, the amendment of diet and other particulars of faulty regimen is indispensable and alike, but for each some distinctive medical procedures are afterwards requisite which are not equally applicable to all. Indeed, we suppose that Dr. Smyth, notwithstanding he considers these affections identical, would scarcely recommend the condition of the liver to be the grand and primary object of attention in scrofula and in hydrocephalus as he does in rickets. It is well known that in most cases of rickets this organ is much enlarged, and in many, the mild alterative aperients which he recommends, the hydr. c. cretâ with jalap, and sulphate of potass and bicarbonate of soda, may often be useful in aiding in the due regulation of its functions; but we are convinced that the treatment of scrofula by mercurials as a general rule and leading object, would lead to as much mischief among children as the too general employment of calomel and blue pill in various forms of indigestion does among adults. It is somewhat strange that the author, who believes scrofula and rickets to be almost or quite identical diseases, considers *tabes mesenterica* and rickets as quite distinct affections in their origin and symptoms. This is a nicety of distinction we are quite unable to appreciate. He believes that the enlarged belly of diseased children is often attributed to diseased mesenteric glands, when it really arises from an increased size of the liver. This is doubtless the case sometimes, if the examination which is made be too superficial; but we suppose that these instances are comparatively rare, as post-mortem examinations far more frequently exhibit an enlarged condition of these glands than of the liver.

Dr. Smyth speaks highly in praise of the regimen recommended for scrofulous children by Sir Astley Cooper, consisting of an abundant supply of animal food, warm clothing, free exercise, and alterative medicines. "There is an error, in my opinion, abroad, of no little evil operation, in some instances, as regards the use of animal food. With a great many individuals, notwithstanding what the advocates of vegetable and farinaceous aliment may say to the contrary, this article of diet is unquestionably taken too sparingly, or its use is not properly timed. The presence of animal food in the stomach at an early period of the day, acts, with many persons of peculiar temperament and constitution, not only as an efficient remedy in soothing and allaying general irritability, and vascular overaction dependent thereon, but it also performs the part of an anodyne and agent of cure in various dyspeptic neuralgias and irritations." Of this there can be no doubt; but when animal food is prescribed three times a day as invariably the best means of counteracting tendency to a scrofulous condition of the economy, we must bear in mind the wise cautions delivered to us by Sir James Clark: for although such regimen would undoubtedly be the best for many feeble children in the lower classes if they could obtain it, it is no less true that those of the upper classes, in families in which scrofula has prevailed, frequently are brought into a diseased condition by an undue supply of animal food furnished to them through the fears of their parents having been excited by the observation of the ill-effects of an under-fed condition. The disease may thus in fact be engendered by two opposite means, both of which equally, although in different ways, disorder and enfeeble the powers of digestion.

We quote part of some not very intelligible remarks upon the circulation.

"Frequently, as may be observed, when the pulse is beating rapidly, and from this, with other relative symptoms, one might be led to infer that the circulation was in a state of hurry and acceleration, it is not so, but just the reverse obtains —the blood is moving slowly, while the heart and arteries, the containing vessels, are acting inordinately. Such discordance in the motion of the blood and its active circulatory apparatus constitutes a condition of disorder frequently met with, of *false* excitement—*excitement with debility*. It is more hardness or resistance, I conceive, in the pulse, together with fulness, and not a high number of beats in a given time, which indicate and announce a rapid strong circulation; and the best ordinary method of nicely appreciating these qualities in the circulation is, when the fingers, in feeling the pulse, have remained some time upon it, to slightly raise them, and gently repress the vessel. * * * * * I have an unsettled opinion, too, that the motion of the blood in the child is slower than in the adult, although its pulse is always considerably more frequent." 60.

*Chronic Hydrocephalus.*—A case is related at very great length in which the operation of tapping was performed upon a child a twelvemonth old, and repeated nine times in the next four months. The quantity of water removed varied from four to forty ounces, amounting in all to 143 oz.—sixty-four additional ounces being found in the head after death. Some amendment of symptoms followed the first six operations, although the water rapidly re-collected in increased quantities, but the latter ones were only performed in the hope of palliating some of the most distressing symptoms. The success which attended Dr. Conquest's operations* does not seem to have been obtained by other observers; and, judging from the results furnished by Dr. West's interesting analysis of cases,† we cannot doubt that the operation will ere long fall into disuse. The author thus alludes to what he considers may be one cause of failure, having previously observed that no marks of inflammatory action were found in the vicinity of the punctured portions of the brain.

"These facts have induced me to think that, perhaps, in this case, it was not so much the puncturing of the brain as the consequent collapse of the organ which gave rise to its several attacks of inflammation; and this opinion, it may be allowed, receives more than a little corroboration by my having observed, during the treatment, that the symptoms of cerebral inflammation and the state of cerebral collapse were always concomitant; that, after each operation, although the brain necessarily became more or less collapsed, if that state continued longer than 24 hours, it was afterwards accompanied by general febrile excitement, and symptoms of cerebral inflammation, and that on the disappearance of these, the dropsical effusion was again quickly repeated, and the brain rendered distended and tense. Such phenomena I observed not only once, but three or four times; and the mode in which it would occur to me to explain them would be, that, under the condition of collapse, the mass and weight of the brain, instead of being supported and pressing, as in the healthy state of the organ is the case, on the sides as well as on the floor of the cavity of the cranium, pressed wholly on this latter part, thereby producing more or less obstruction of the circulation, with some degree of irritation of the interposed membranes, on which the inflammatory attacks supervened; that, during the existence of these attacks, and more especially of the sympathetic febrile excitement, owing to, and concomitant with, the *general* arrest of secretion and exhalation which accompanies and characterizes such a state of the system, the *local* effusion in the present instance was suspended, and the brain, in consequence, remained collapsed. But, as has already been remarked, as these inflammatory and febrile states disappeared, and the processes of exhalation and secretion generally resumed activity, the dropsical effusion was speedily repeated, and the brain rendered distended and tense." 135.

Dr. Smyth recommends, therefore, that in future operations collapse should be prevented by limiting the quantity of water extracted at one time to from four to six ounces.

*Cerebral Auscultation.*—Unaware that the phenomenon had been observed previously by some of the physicians of the United States, Dr. Smyth described in 1837 the results of auscultation applied to the heads of rickety and hydrocephalic children, and subsequent experience induces him to believe, that a certain sound precedes and accompanies dropsical effusion into the cranium.

"It is an abrupt, brief, rushing, arrested sound, in tone something between a *bruit de soufflet* and a *bruit de rape;* not soft enough for the former, nor hard enough for the latter. In its character of intensity it varies, of course, with the

---

* See Medico-Chir. Rev. July 1838, p. 273.
† Med. Gazette, xxx. 127.

energy of the action of the heart and pulse. When the circulation is excited and vigorous, and the heart, unembarrassed by palpitation, beats steadily and strongly, the sound is most clear and audible."

The author observes that, although the subject has not been followed up effectually in this country, it has met with better success in America. Dr. Whitney enumerates the following conditions, in which the cerebral murmur can be heard: viz. simple congestion of the brain; acute inflammation with or without effusion; chronic hydrocephalus; local compression of the brain; ossification of its arteries; induration of its substance; aneurism of a cerebral artery; certain hydrocephaloid diseases and states of excessive anæmia and exhaustion of the system, *e. g.* severe chlorosis and amenorrhœa. "In cases of chlorotic cachexia and severe general and cerebral anæmia, the cerebral murmur or bellows-sound is observed to become modified in its character, and to acquire some tones similar to those that are blended with the respiratory murmur in bronchitis. It acquires musical, cooing, chick-like tones. In aneurism of a cerebral artery it puts on a thrilling, purring character." Dr. Whitney further states that the *voice*, as heard by applying the ear to the skull, undergoes remarkable changes.

"This last sound in certain cases of cerebral inflammation and effusion between the membranes and on the surface of the brain, has been observed to undergo a change of character, similar to that which it undergoes in its passage through the lung and wall of the chest in some cases of pleuritis and pleuritic effusion. It acquires an ægophonic tone, which, according to the American writers, may be considered as a certain diagnostic sign of effusion between the membranes of the brain. Thus, it would appear, that we have a cephalic ægophony expressive of a particular condition of disease of the brain, as we have a thoracic ægophony expressive of a particular condition of disease of the chest." 151.

*Impotence.*—In the more than one hundred pages which the author devotes to this subject, we can discover little or nothing of novelty or interest. We believe cases, similar to those which he details with too great prolixity, are continually falling under the notice of every well-employed practitioner. That impotence may depend upon a generally disordered or diseased condition of the economy, rather than upon any particular lesion of the generative organs, is well known; and probably every medical man would seek, as the author did, to restore health by the due administration, first of aperients, alteratives, stomachics, &c. &c., and then of chalybeates, and by attention to diet and other points of regimen, rather than by any attempt at the employment of any more specific treatment. So, too, in those cases, constituting the majority, in which impotence results from the excessive or perverted employment of the sexual organs, and is, as it always is, accompanied with a shattered condition of the general health. This last becomes the main object of attention, and providing all improper practices are discontinued, when it is amended, we frequently find the function of generation restored. All this, which, as well as the vast depressing influence this class of disorders exerts upon the mind, the author seems to announce almost as a novelty, has been long known to, and is continually acted upon, by every well-educated medical man. The misfortune is, however, that a diseased condition of the economy, which perhaps years of misconduct have entailed upon the sufferer, requires at least months of a carefully adapted and diligently pursued course of medical treatment to produce any alleviation, and a still longer period to prevent the likelihood of a relapse. The patient soon gets tired of this, and flies from practitioner to practitioner in the hopes of finding a readier road to restoration, until, at last, he throws himself in despair into the arms of quackery, which promises him every thing he requires, and, by the influence upon the mind which bold assertions, that no respectable practitioner would hazard, produce, such promises even may sometimes be realized. Few practitioners of credit have these cases long enough under their care for the full operation of the remedies prescribed; and some of those which the author concludes were cured by the judicious means he recommended, because they did not apply to him again, may probably have sought assistance elsewhere.

While upon this subject, we may observe that the manner in which some of the cases are narrated is somewhat unusual for publications intended solely for the profession, and gives the work somewhat of an appearance which we feel sure the author never intended it should put on. Thus the symptoms and progress of the case are often related by the patients themselves, just as we find they are in empirical publications. For example, one begins—" Sir, I have been induced, by the perusal of your paper in a number of the Lancet, to seek your opinion and advice. An abject and degraded being, (for whom language has no name,) I could not consult you personally, and feel that I owe you an apology for addressing you on such a subject. * * * I am, Sir, your obedient servant, H. I. Address Post-office, C——h street." Again—" Sir, I have lately taken up a number of the Lancet, in which it appears that you have treated successfully that disease which I had almost given up in despair as absolutely incurable, namely, a case of impotence, arising, to a certain extent, from early depraved use of the genital power. Pray advise me under similar circumstances. * * * I am, Sir, your obedient servant, R. S. I. Post-office, C——t street." And then we have repeated notes detailing the various symptoms, effects of remedies, &c. Now, when we consider that these papers appeared originally in the Lancet, a work which professes to circulate much among the lay-public, we think this graphic style of narration might advantageously have been exchanged for one more simple, and certainly more usual, quite as convincing, and involving much less repetition of details, which at the best do not form the most agreeable reading.

Dr. Smyth believes that the intimate connexion of a diseased condition of the generative organs and disorders of the intellect is not sufficiently appreciated.

" The constant association of sexual disorder, and more or less of generative incapacity, with mental derangement, whether as cause or effect, is a remarkable fact, and one which appears to me not to be very generally known ; yet I will venture to say, that every insane individual, whether male or female, is at the same time also the subject of some sort of procreative disability, defect, or disorder, either impotence or sterility, or both; and the removal of the one affection (most frequently, I apprehend, the mental) would often seem to prove immediately curative of the other. I have seen several instances of insanity accompanied by impotence and barrenness in both sexes, in which such, certainly, appeared to have been the case, in which, by the adoption of a similar mode of treatment in each case, the return of reason, and the resuscitation of the sexual powers, were so strictly concomitant, that it was impossible not to infer but that either the one disease was the cause of the other, or that both affections depended upon one common cause." 173.

One of the offices of the seminal fluid is thus stated :—

"The spermatic fluid, which every one knows it is the office of the testicles to secrete, every one should at the same time be aware is not, as it is too commonly supposed, an excrementitious fluid, and intended, like the urine, to be eliminated from the body; but, on the contrary, (except during an occasional act of generation), to be received into the circulation, and thence distributed to every part of the system. It is the presence of the semen in the circulatory fluids of the male, and the accumulated influence of unexhausted ovaria in the system of the female, which gives to the countenances of the continent and chaste the peculiar expression of energy and vigorous health which generally characterise them, and which, though the features themselves should not be fashioned to the lines of beauty, never fails, notwithstanding, to impress the beholder with a sense of admiration, and some feeling of respect." 177.

Dr. Smyth sets a high value upon Plummer's pill, as an alterative after due purgation in certain cachectic conditions of the economy.

"In cases in which the pathology is not a thing of morbid relievo, positive, and substantial, but more a condition the reverse, a condition altogether of degree of physiological imperfection, the proximate causes of the affections consisting, for the most part, in weak and languid circulation generally, and tardy and defective performance of all the vital functions—Plummer's pill, as an alterative, rightly timed and apportioned, is without an equal. It seems to me to enliven and excite, in a peculiarly salutary manner, all the nutritive actions of the economy, and also to leave upon these, after its administration has been discontinued, a somewhat persistent impression of its virtue. No simple or compound medicinal substance, that I am acquainted with, possesses the power to such a degree, of developing animal heat generally throughout the system, without the weakening accompaniment of perspiratory moisture; on which account, perhaps, it is that I have found it an exquisite tonic and deobstruent, in cases of dyspepsia, of general cachectic debility, and of amenorrhœa, in persons of cold phlegmatic temperament. Sometimes alone, and occasionally in more or less quantity in union with myrrh and aloes, (as in the pill of aloes with myrrh of the Pharmacopœia,) I have found it successful in the cure of amenorrhœa, after chalybeates and a variety of other emmenagogues had been tried in vain. I have also witnessed its good effects in the cold species of chronic rheumatism. It is, unquestionably, I think, the mildest and most delicate alterative we have." 215.

